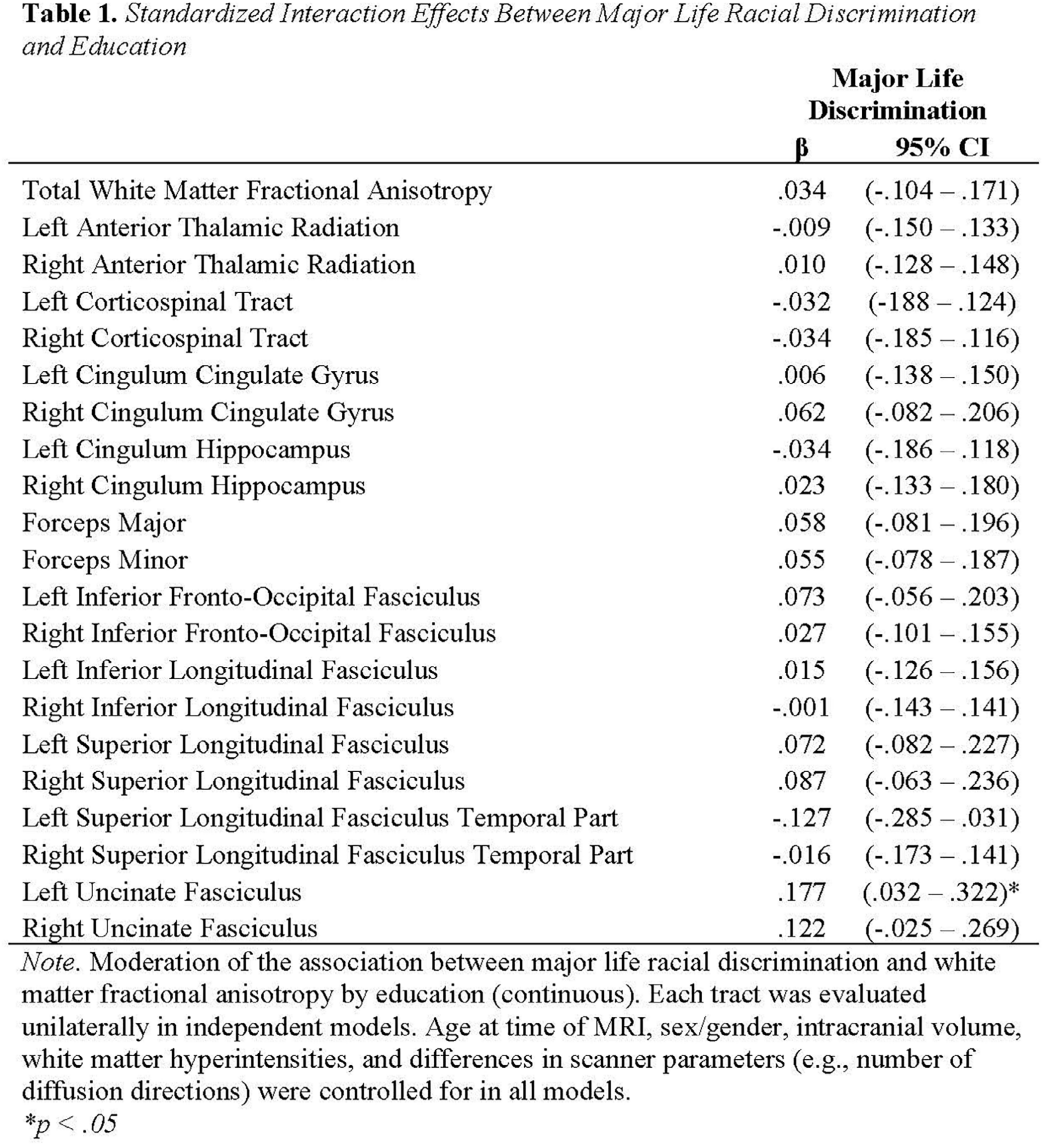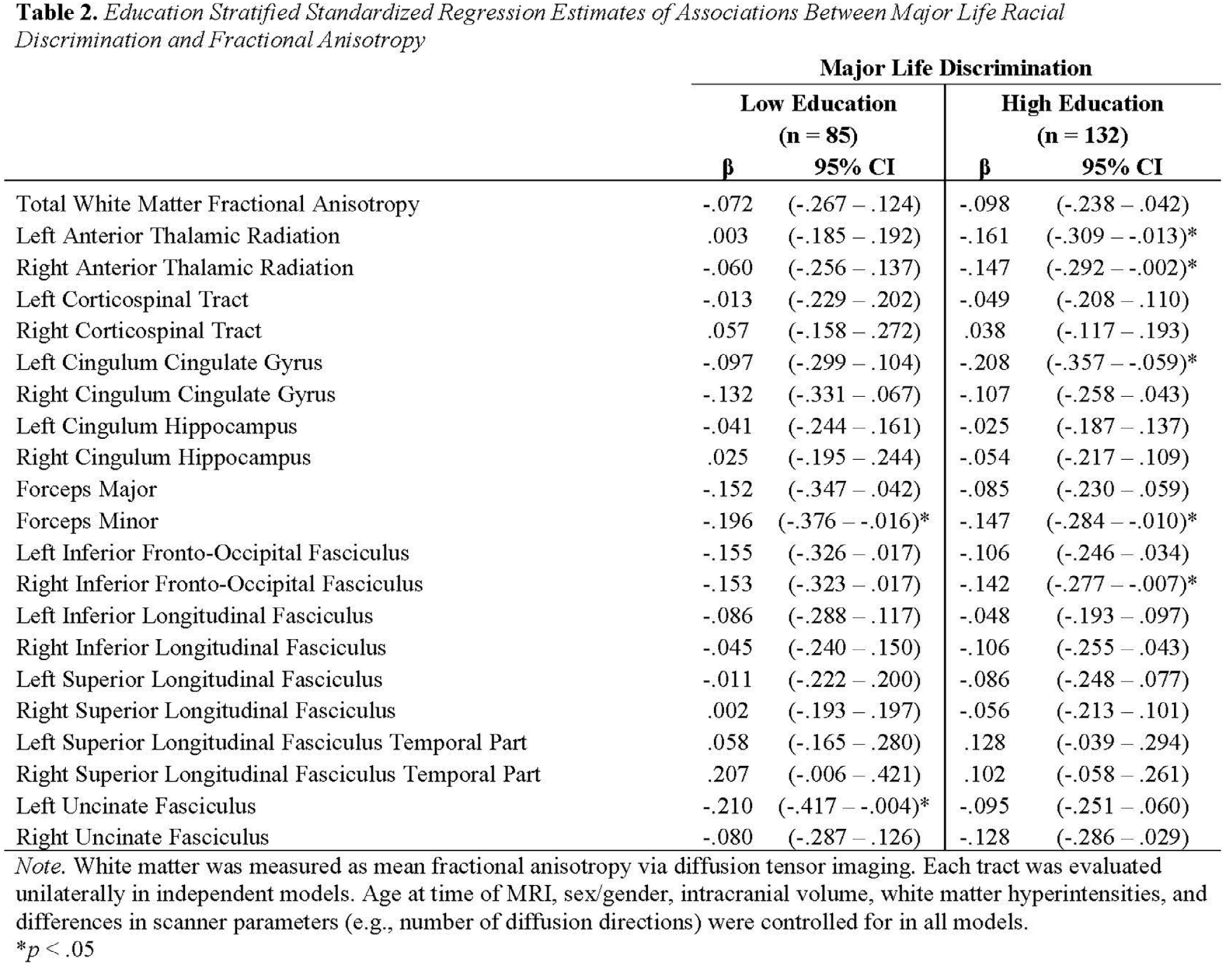# The Impact of Education on the Relationship Between Racial Discrimination and White Matter Among Black Older Adults

**DOI:** 10.1002/alz70860_098808

**Published:** 2025-12-23

**Authors:** Jordan D. Palms, Emily P. Morris, Kiana A. Scambray, Ketlyne Sol, Clarissa Morales, Robrielle M. Pierce, Monica E. Walters, Patrick J. Lao, Jennifer J. Manly, Adam Brickman, Laura B. Zahodne

**Affiliations:** ^1^ University of Michigan, Ann Arbor, MI, USA; ^2^ Taub Institute for Research on Alzheimer's Disease and the Aging Brain, New York, NY, USA; ^3^ Department of Neurology, Gertrude H. Sergievsky Center and the Taub Institute for Research on Alzheimer's Disease and the Aging Brain, New York, NY, USA

## Abstract

**Background:**

Racial discrimination among Black older adults is negatively associated with measures of white matter health (e.g., fractional anisotropy [FA]), which serve as important indicators of healthy aging. However, the effect of discrimination may differ by socioeconomic position. Higher levels of education may increase exposure to settings with a greater likelihood of discrimination but decrease vulnerability to negative health outcomes. We evaluated the relationship between discrimination and white matter FA at different levels of education. We hypothesized that the relationship between discrimination and FA would be weaker in people who have higher education due to increased access to resources.

**Methods:**

Black older adults from the Washington Heights‐Inwood Columbia Aging Project who underwent MRI and completed psychosocial questionnaires were included (*N* = 217). Racial discrimination (i.e., civil rights violations) was measured with the Major Life Experiences of Discrimination Scale. Total brain and regional FA were collected via diffusion weighted MRI. Linear regressions for whole brain and individual tracts (*N* = 20) were performed. Interaction terms tested whether education moderated associations between discrimination and FA. Subsequent models were stratified by education: high school or less vs. greater than high school. Covariates included age, sex/gender, intracranial volume, white matter hyperintensities, and scanner parameters (e.g., number of diffusion directions).

**Results:**

Black older adults with higher education reported more racial discrimination‐based civil rights violations (t(215)=3.63, *p* < .001, Cohen's d=.51) than those with lower education. More discrimination was associated with lower FA in the left uncincate fasciculus among those with lower education, but not among those with higher education (significant discrimination by education interaction). However, in stratified models there was a negative association between discrimination and FA in bilateral anterior thalamic radiation and left cingulate cingulum gyrus that was larger among those with higher education. Associations involving other tracts were similar across education groups (e.g., forceps minor, right inferior fronto‐occipital fasciculus).

**Conclusions:**

FA appears to act as a mechanism in which discrimination impacts brain health via tracts involved in social‐emotional processing. Individuals with higher education reported more experiences of discrimination in this sample. Higher education may buffer against discrimination for some tracts while a threshold effect may exist for other tracts.